# Repeatability of radiomic features in myocardial T1 and T2 mapping

**DOI:** 10.1007/s00330-024-11337-8

**Published:** 2025-01-15

**Authors:** Mathias Manzke, Fabian C. Laqua, Benjamin Böttcher, Ann-Christin Klemenz, Marc-André Weber, Bettina Baeßler, Felix G. Meinel

**Affiliations:** 1 Institute of Diagnostic and Interventional Radiology, Pediatric Radiology and Neuroradiology, University Medical Centre Rostock, Rostock, Germany; 2 Department of Diagnostic and Interventional Radiology, University Hospital Würzburg, Würzburg, Germany

**Keywords:** Radiomics, Cardiac magnetic resonance imaging, T1 mapping, T2 mapping, Reproducibility

## Abstract

**Purpose:**

To investigate the test–retest repeatability of radiomic features in myocardial native T1 and T2 mapping.

**Methods:**

In this prospective study, 50 healthy volunteers (29 women and 21 men, mean age 39.4 ± 13.7 years) underwent two identical cardiac magnetic resonance imaging (MRI) examinations at 1.5 T. The protocol included native T1 and T2 mapping in both short-axis and long-axis orientation. For T1 mapping, we investigated standard (1.9 × 1.9 mm) and high (1.4 × 1.4 mm) spatial resolution. After manual segmentation of the left ventricular myocardium, 100 radiomic features from seven feature classes were extracted and analyzed. Test–retest repeatability of radiomic features was assessed using the intraclass correlation coefficient (ICC) and classified as poor (ICC < 0.50), moderate (0.50–0.75), good (0.75–0.90), and excellent (> 0.90).

**Results:**

For T1 maps acquired in short-axis orientation at standard resolution, repeatability was excellent for 6 features, good for 29 features, moderate for 19 features, and poor for 46 features. We identified 15 features from 6 classes which showed good to excellent reproducibility for T1 mapping in all resolutions and all orientations. For short-axis T2 maps, repeatability was excellent for 6 features, good for 25 features, moderate for 23 features, and poor for 46 features. 12 features from 5 classes were found to have good to excellent repeatability in T2 mapping independent of slice orientation.

**Conclusion:**

We have identified a subset of features with good to excellent repeatability independent of slice orientation and spatial resolution. We recommend using these features for further radiomics research in myocardial T1 and T2 mapping.

**Key Points:**

***Question*** *The study addresses the need for reliable radiomic features for quantitative analysis of the myocardium to ensure diagnostic consistency in cardiac MRI.*

***Findings*** *We have identified a subset of radiomic features demonstrating good to excellent repeatability in native T1 and T2 mapping independent of slice orientation and resolution.*

***Clinical relevance*** *Radiomic features have been proposed as diagnostic and prognostic biomarkers in various heart diseases. By identifying a subset of particularly reproducible radiomic features our study serves to inform the selection of radiomic features in future research and clinical applications.*

**Graphical Abstract:**

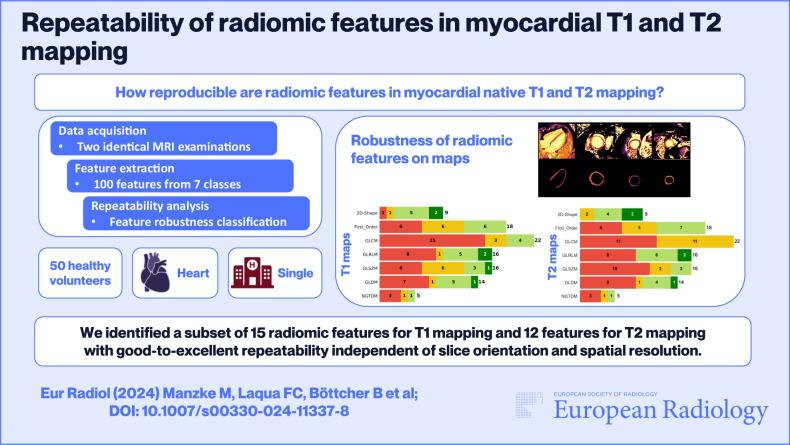

## Introduction

Cardiac magnetic resonance imaging (MRI) mapping sequences allow for quantitative tissue characterization of the myocardium [1]. Myocardial relaxation times reflects biophysical properties of the myocardial muscle cells and the surrounding extracellular space. Quantifying T1 or T2 relaxation times is therefore helpful to diagnose pathologies affecting the myocardium. Altered myocardial relaxation times can result from fibrosis, inflammation, edema or storage diseases [2].

In addition to evaluating mean global or regional T1 and T2 relaxation times, radiomics features have been used to extract detailed quantitative information from myocardial T1 and T2 maps. Radiomics features are essentially numerical data extracted from segmented regions of interest (ROI) within radiological images that capture a variety of aspects regarding image texture, shape, intensity, and patterns and may serve as imaging biomarkers of myocardial disease [3, 4]. Radiomics analysis of myocardial native T1 mapping has been used as diagnostic and prognostic biomarkers in a wide range of cardiac conditions. T1 mapping radiomics features have been investigated as diagnostic biomarkers to diagnose myocarditis [5], characterize the extracellular matrix in dilated cardiomyopathy [6], classify hypertrophic cardiomyopathy genotypes [7] and phenotypes [8, 9], distinguish hypertrophic cardiomyopathy from hypertensive heart disease [10, 11] and differentiate cardiac tumors from thrombi [12]. As prognostic biomarkers, T1 radiomics features have been suggested to predict clinical course in dilated cardiomyopathy [13, 14], heart failure in systemic lupus erythematosus [15], functional recovery after myocardial infarction [16], and treatment response in pulmonary arterial hypertension [17]. The application of radiomics analysis of T2 maps has focused on the diagnosis of acute and chronic myocarditis [5].

An essential prerequisite for diagnostic or prognostic significance is the repeatability of the measurement results. Although the reproducibility of radiomics features has received significant attention [18–20] and has been studied for cardiac cine sequences [21], there is very limited data on the repeatability of radiomics features in myocardial T1 and T2 mapping. Only one previous study specifically investigated the reproducibility of myocardial T1 and T2 radiomics and found that only a small subset of myocardial radiomic features were reproducible [22]. This previous analysis focused on the overall performance of feature classes, not individual features. The influence of slice orientation (short-axis vs. long-axis view) and spatial resolution was also not investigated.

Therefore, the aim of this study was to systematically analyze the repeatability of individual radiomic features in myocardial native T1 and T2 mapping.

## Material and methods

### Patient selection and study design

We performed a post-hoc analysis of data from a prospective study of 50 healthy participants (29 female, 21 male, mean age 39.4 ± 13.7 years), without cardiovascular disease. Mean BMI was 23.9 ± 4.0 kg/m^2^, and the mean heart rate was 72 ± 10 per minute, who underwent two identical cardiac MRI examinations. The distribution of demographic parameters is shown in Suppl. Fig. 1. All MRI examinations were performed on a 1.5-T MRI scanner (Avanto Fit Siemens Healthineers). Details of imaging protocols and the process of creating the T1 maps have been published previously [23].

### Ethical approval and informed consent

This study was approved by the institutional review board (Ethics Committee, University Medical Center Rostock), and written informed consent was obtained from all volunteers prior to enrollment. The study conformed to the ethical guidelines of the Helsinki Declaration (revised version from 2013).

### Image acquisition

Each volunteer underwent two MRI investigations on the same day with a break of at least 20 min between the examinations. All MRI examinations were performed on a 1.5-T MRI scanner (Avanto Fit, Siemens Healthineers). For T1 and T2 mapping, three short-axis (SAX) slices were acquired in a basal, midventricular, and apical position of the left ventricle. Additionally, long-axis T1 and T2 mapping was performed in the four-chamber view (4Ch). For T1 mapping, a standard MOdified Look-Locker Inversion recovery (MOLLI) 5(3)3 sequence was used and acquired in two different spatial resolutions: standard resolution with 1.9 × 1.9 mm pixel size and high resolution with 1.4 × 1.4 mm pixel size. Further details of the imaging protocol have been published previously [23, 24].

### Image processing

The left ventricular myocardium was manually segmented on all mapping data by one radiologist using open-source software (ITK-SNAP, version 3.8.0). Segmentation was performed on the original source images, not scanner-generated maps. We deliberately chose to exclude a thin subendocardial and subepicardial layer of the myocardium to avoid any risk of inadvertently including the left ventricular blood pool or epicardial fat in the segmentation.

### Radiomics feature extraction

Radiomic features of the left ventricular myocardium were extracted using open-source Python packages (pyradiomics, version 3.0.1, SimpleITK 2.0.2, numpy 1.21.5) for all segmentations of the T1 and T2 mapping data. We analyzed all 100 radiomic features as provided by the software from the following seven feature classes: First Order Features, Shape Features (2D), Gray Level Co-occurrence Matrix (GLCM) features, Gray Level Size Zone Matrix (GLSZM) Features, Gray Level Run Length Matrix (GLRLM) features, Neighboring Gray Tone Difference Matrix (NGTDM) features and Gray Level Dependence Matrix (GLDM) features (Table 1 and Supplementary Material).

### Statistical analysis

Test–retest repeatability was assessed by using the intraclass correlation coefficient (ICC) as the primary parameter according to the definition of Shrout and Fleiss [25]. This criteria represents the feature robustness and, hence, the reproducibility [26]. For all radiomic features, ICCs were calculated with 95% confidence intervals from two-way random effects for the models of both measurements. Numerical ICC values were interpreted as follows: < 0.50: poor, 0.50–0.75: moderate, 0.75–0.90: good, > 0.90: excellent [27].

To further assess the reproducibility, we used the concordance correlation coefficient (CCC) [28, 29]. The Pearson correlation coefficient *r* was calculated to measure the linear relationship between both measurements.

Detailed results for all features were presented as tables for standard resolution and short-axis orientation as these can be considered the clinical standard. We further analyzed results also for long-axis T1 and T2 mapping (4Ch) and for high-resolution T1 mapping. For each of these settings and each of the seven feature classes, the number of features with excellent, good, moderate, and poor reproducibility was visualized as stacked bar charts.

We further aimed to identify a subset of radiomic features with good to excellent repeatability regardless of image orientation and spatial resolution. For T1 mapping, we identified the intersection of features that showed good to excellent repeatability (ICC > 0.75) in all four sequence variations: short-axis and long-axis views as well as standard and high spatial resolution. For T2 mapping, we determined the intersection of features with good to excellent repeatability (ICC > 0.75) in short-axis and long-axis views. The resulting selections of robust radiomic features were visualized and color-coded according to their ICC values over all inhabited feature sets. For these features, we further analyzed for differences in their numerical values between female and male participants as well as younger and older participants. For this purpose, the measured values were clustered according to gender and age group, and then the mean value was determined for each characteristic over both runs. The nonparametric Mann–Whitney *U*-test was used to test for gender- or age-specific differences.

**Fig. 1:**
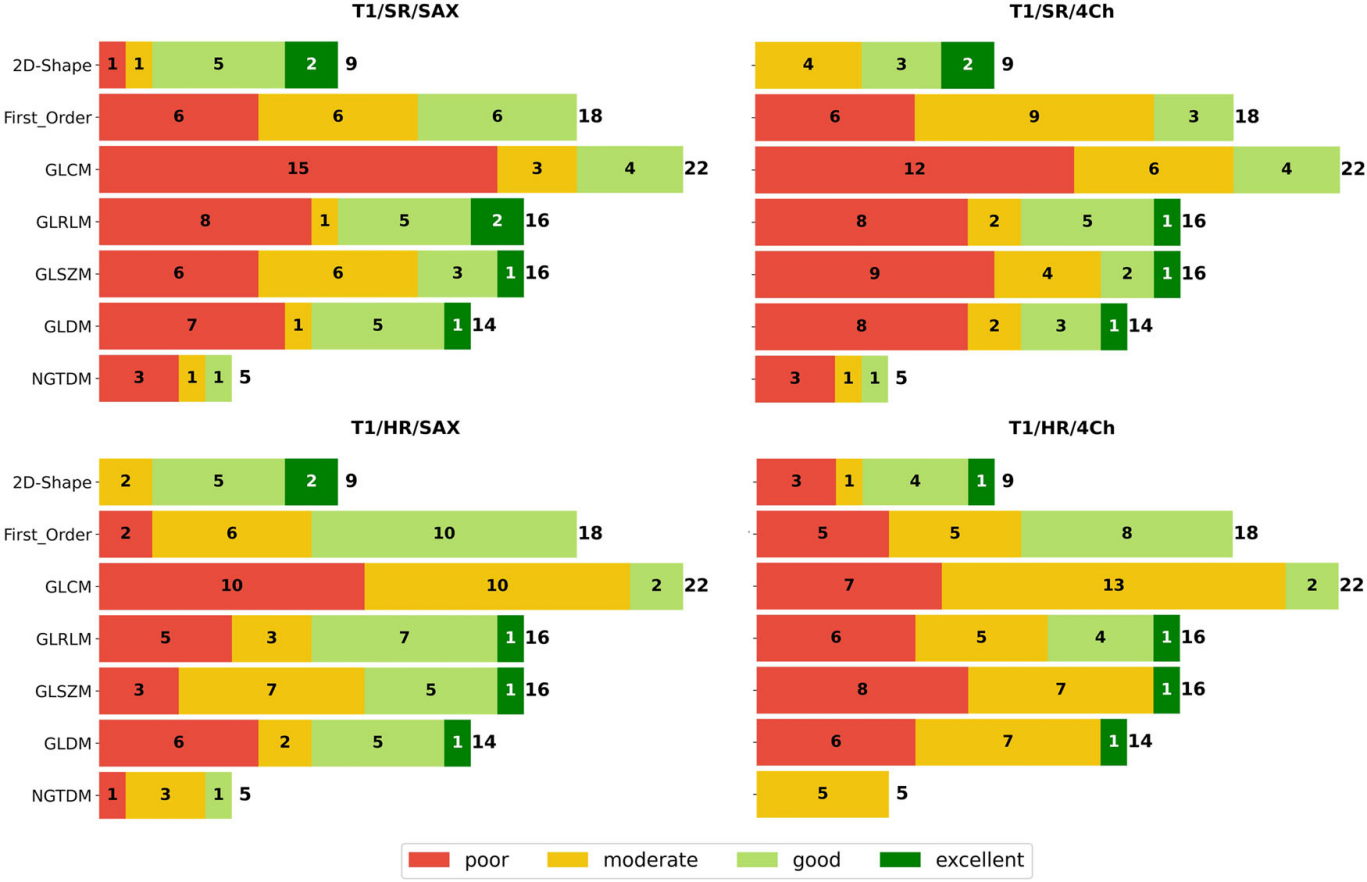
Robustness of radiomic feature classes for T1 mapping: for all combinations of short-axis (SAX) or four-chamber view (4Ch) and high resolution (HR) or standard resolution (SR) T1 mapping, the figure shows the distribution of robustness for all seven radiomic feature classes considered: Shape Features (2D), First Order Features, Gray Level Co-occurrence Matrix (GLCM) features, Gray Level Size Zone Matrix (GLSZM) Features, Gray Level Run Length Matrix (GLRLM) features, Neighboring Gray Tone Difference Matrix (NGTDM) features and Gray Level Dependence Matrix (GLDM). Green = excellent (ICC > 0.9), light green = good (ICC 0.75–0.90), yellow = moderate (ICC 0.5–0.75), red = poor (ICC < 0.5) robustness

**Fig. 2:**
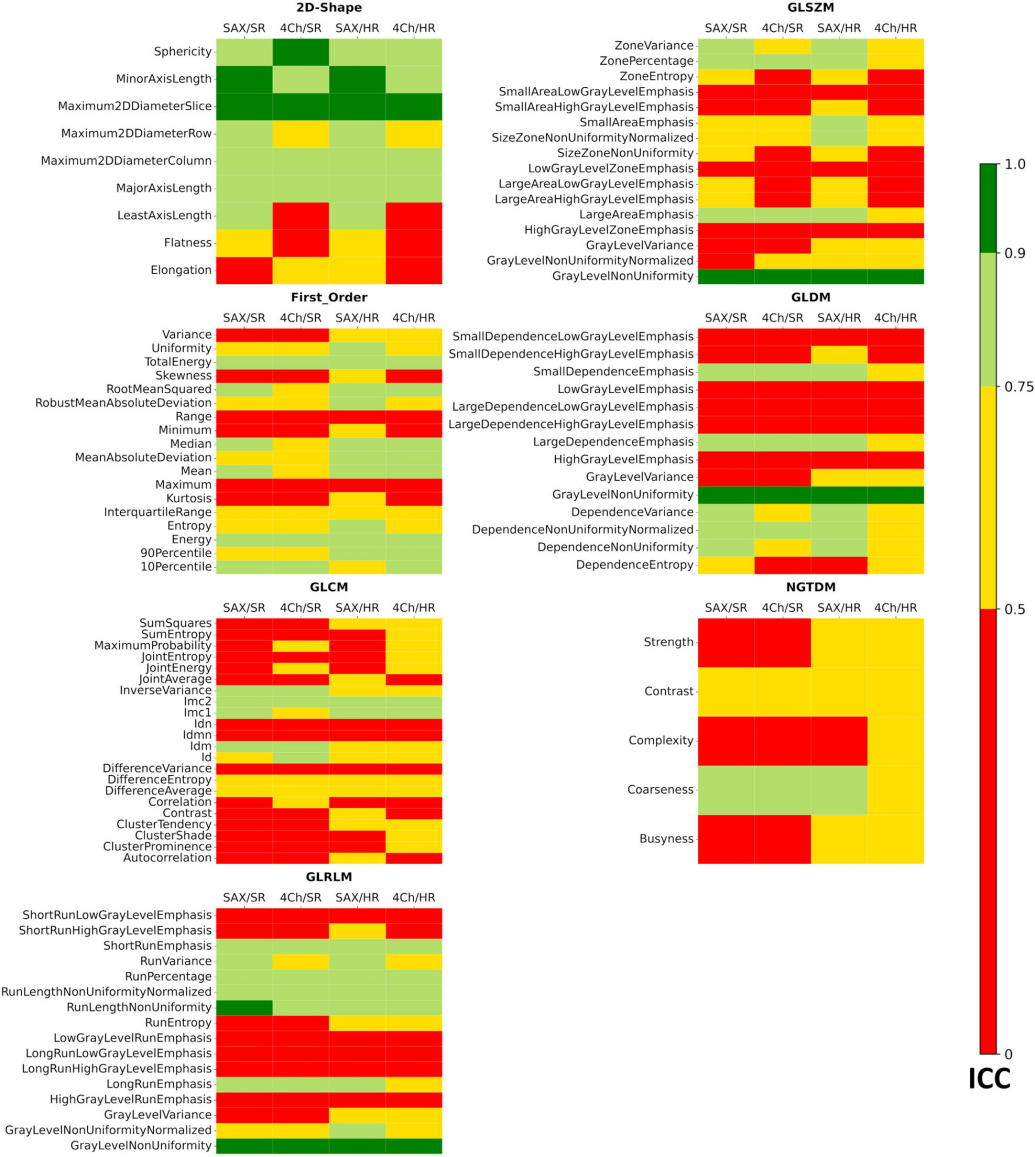
Robustness of all radiomic features for T1 mapping: for all combinations of short-axis (SAX) or four-chamber view (4Ch) and high resolution (HR) or standard resolution (SR) T1 mapping, all features are color-coded depending on the ICC-value divided for Shape Features (2D), First Order Features, Gray Level Co-occurrence Matrix (GLCM) features, Gray Level Size Zone Matrix (GLSZM) Features, Gray Level Run Length Matrix (GLRLM) features, Neighboring Gray Tone Difference Matrix (NGTDM) features and Gray Level Dependence Matrix (GLDM). Green = excellent (ICC > 0.9), light green = good (ICC 0.75–0.90), yellow = moderate (ICC 0.5–0.75), red = poor (ICC < 0.5) robustness

**Fig. 3:**
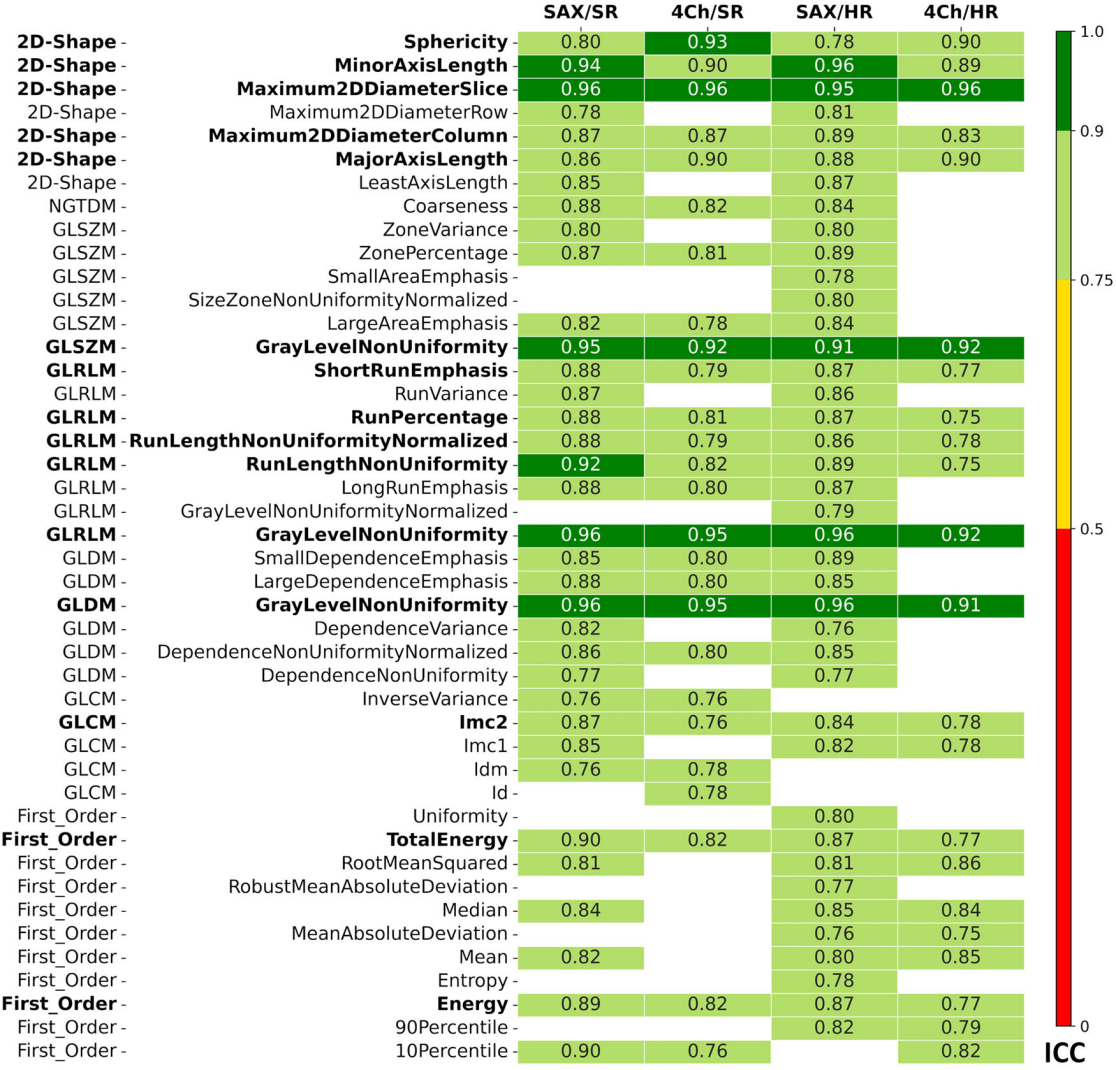
Selected features for T1 mapping: only features with good to excellent reproducibility in at least one of the four sequence variations (short-axis and long-axis views as well as standard and high spatial resolution) are shown. ICC values are color-coded for both views (short-axis/four chambers) and both resolutions. ICC values ≤ 0.75 are not shown. The subset of 15 features that showed good to excellent repeatability (ICC > 0.75) in all four sequence variations is shown in bold

**Fig. 4:**
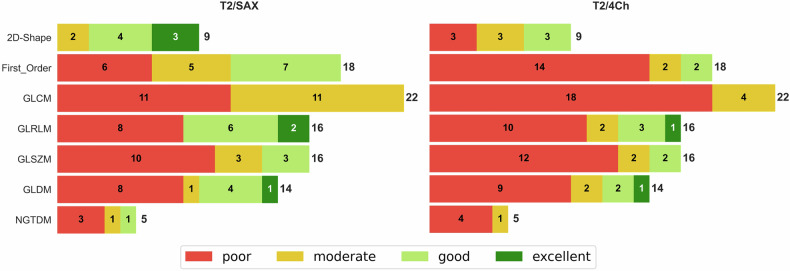
Robustness of radiomic feature classes for T2 mapping: for short-axis (SAX) and four-chamber view (4Ch) T2 mapping, the figure shows the distribution of robustness for all seven radiomics feature classes considered: Shape Features (2D), First Order Features, Gray Level Co-occurrence Matrix (GLCM) features, Gray Level Size Zone Matrix (GLSZM) Features, Gray Level Run Length Matrix (GLRLM) features, Neighboring Gray Tone Difference Matrix (NGTDM) features and Gray Level Dependence Matrix (GLDM). Green = excellent (ICC > 0.9), light green = good (ICC 0.75–0.90), yellow = moderate (ICC 0.5–0.75), red = poor (ICC < 0.5) robustness

**Fig. 5:**
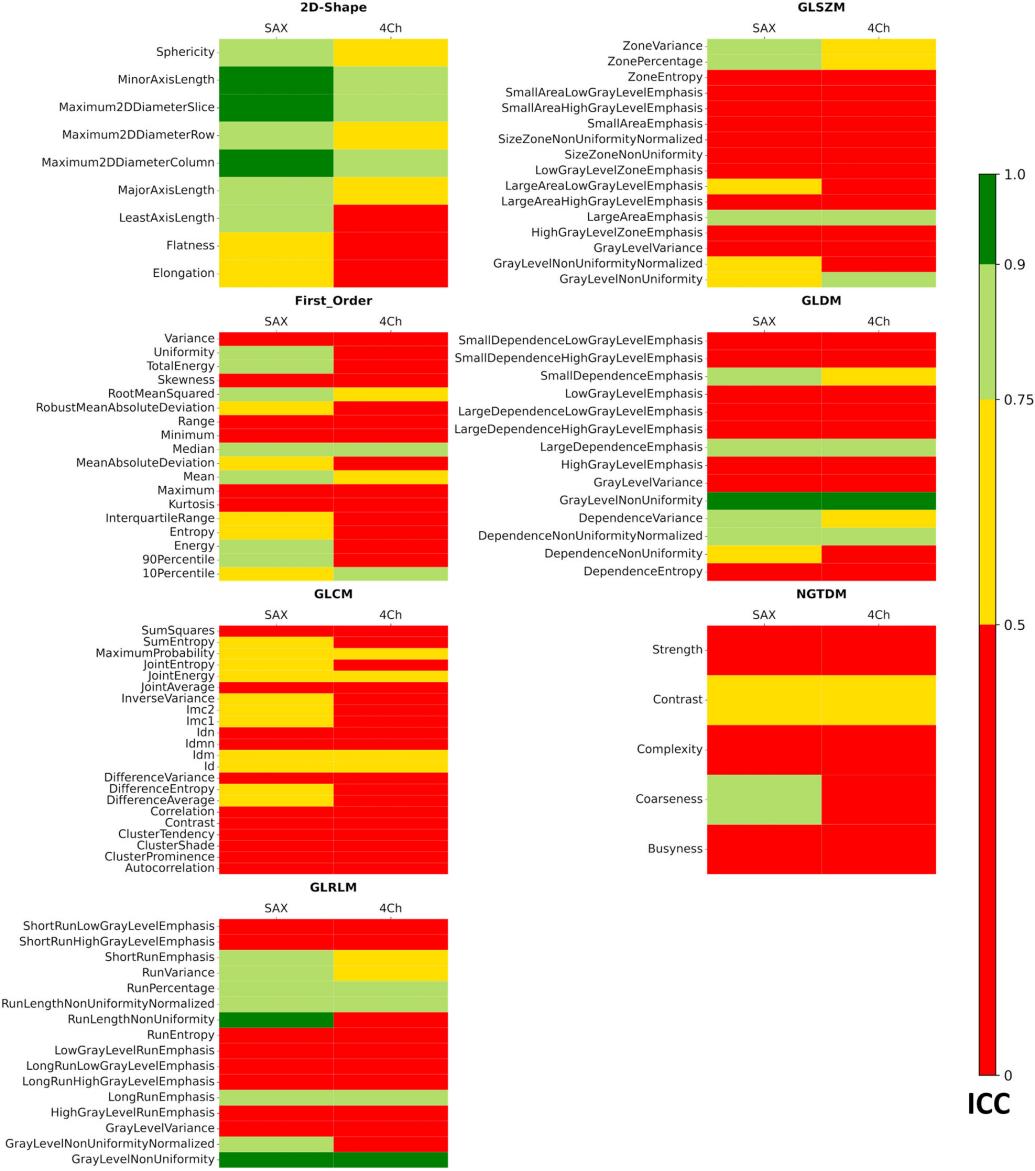
Robustness of all radiomic features for T2 mapping: for short-axis (SAX) and four-chamber view (4Ch) T2 mapping, all features are color-coded depending on the ICC-value divided for Shape Features (2D), First Order Features, Gray Level Co-occurrence Matrix (GLCM) features, Gray Level Size Zone Matrix (GLSZM) Features, Gray Level Run Length Matrix (GLRLM) features, Neighboring Gray Tone Difference Matrix (NGTDM) features and Gray Level Dependence Matrix (GLDM). Green = excellent (ICC > 0.9), light green = good (ICC 0.75–0.90), yellow = moderate (ICC 0.5–0.75), red = poor (ICC < 0.5) robustness

**Fig. 6:**
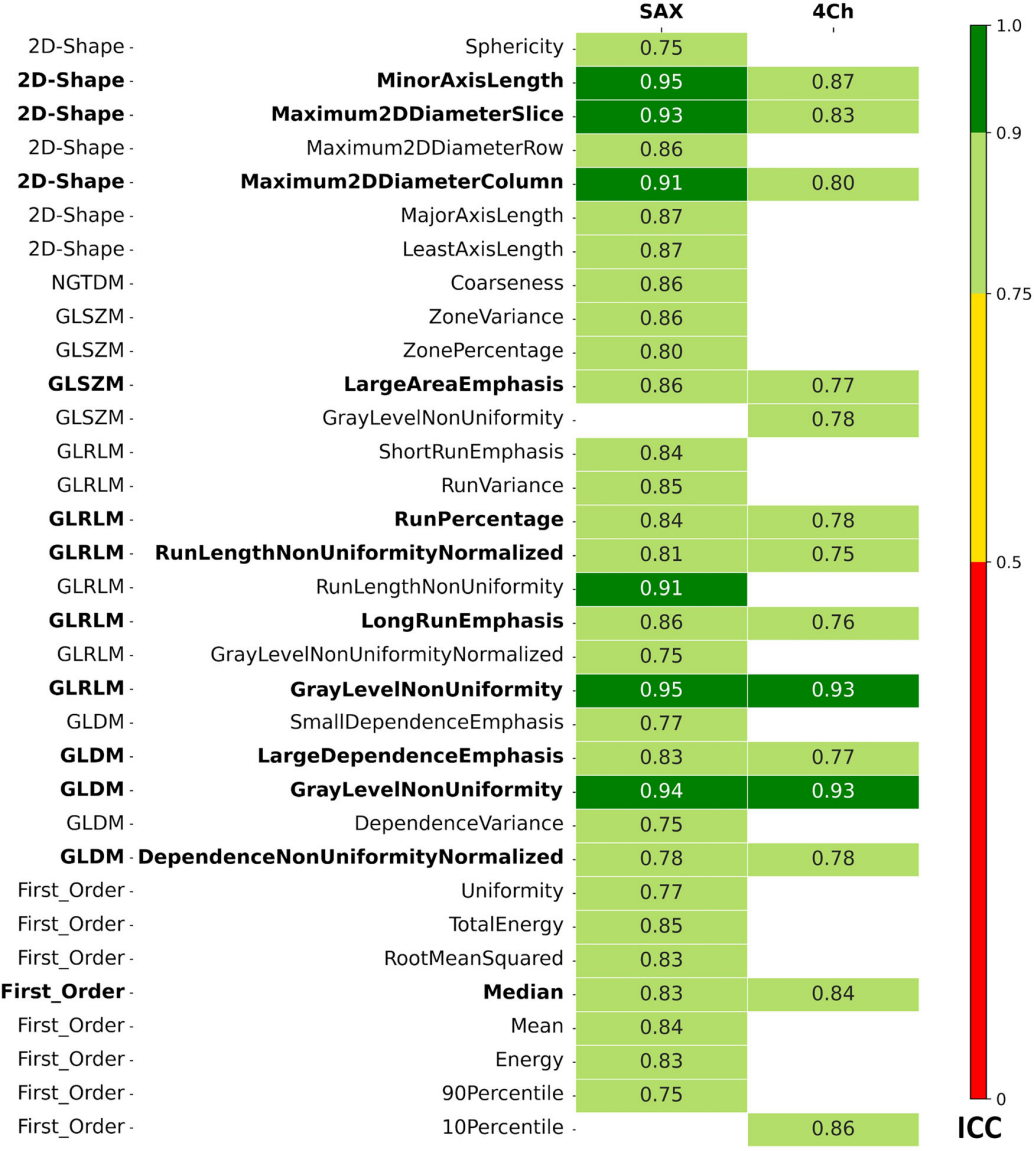
Selected features for T2 mapping: only features with good to excellent reproducibility in at least one of the views (short-axis and 4-chamber view) are shown. ICC values are color-coded for both views (short-axis/4-chamber). ICC values ≤ 0.75 are not shown. The subset of 12 features that showed good to excellent repeatability (ICC > 0.75) in both views is shown in bold

Statistical analyses were performed using the Python (3.12.3) libraries Scipy 1.10.1 for Pearson Correlation Coefficient and Pingouin 0.5.4 for ICC as well as Seaborn 0.12.2. for heatmap plots (Figs. 2, 3, 5, and 6) and Matplotlib 3.9.2 for bar charts (Figs. 1 and 4). Within the Pingouin library, ICC type 2 (single random raters) was used.

## Results

### Repeatability of radiomic features in T1 mapping (standard setting)

T1 mapping acquired with standard (1.9 ×1.9 mm) in-plane spatial resolution in short-axis orientation was considered the clinical standard. For this setting, the repeatability of all radiomic features in each feature class is shown in Supplementary Tables 2–8 (Supplementary Material), sorted in descending order according to their robustness. We observed substantial differences in the repeatability of radiomic features. Across all feature classes, repeatability was excellent for 6 features, good for 29 features, moderate for 19 features, and poor for 46 features. Within each feature class, the repeatability of individual features was highly variable, ranging from poor to good or excellent for all feature classes.

While we used the ICC as the primary parameter of repeatability and robustness, the results for CCC and Pearson correlation coefficient *r* show very consistent results for the robustness of individual radiomic features.

### Influence of image orientation and spatial resolution on the repeatability of radiomic features in T1 mapping

The aggregated results of the entire T1 evaluation are summarized as stacked bar charts in Fig. 1. These charts show the percentage of features with excellent, good, moderate and poor robustness for each feature class and each of the investigated variations of T1 mapping techniques (short-axis and 4Ch in standard and high spatial resolution).

Independent of view or resolution, 2D Shape features were most reproducible, with at least 55.5% of features in this class showing excellent or good reproducibility. The least robust feature classes were NGTDM and GLCM, with 0–20% and 9.1–18.2%, respectively, of features in this class showing excellent or good reproducibility.

The performances of all features are shown in Fig. 2 for all views and all resolutions (Fig. 3) gives a more detailed representation of those features that showed good to excellent reproducibility in at least one of the four sequence variations: short-axis and long-axis views, as well as standard and high spatial resolution. Overall, the reproducibility of radiomic features was superior in the short-axis view (SAX) than in the 4Ch: 32 features from 7 classes showed good to excellent reproducibility in short-axis T1 mapping, compared to only 16 features from 6 classes in the 4Ch (Fig. 3). The number of features with good to excellent reproducibility was 35 for SAX and standard resolution, 26 for 4Ch and standard resolution, 40 for SAX and high resolution and 22 for 4Ch and standard resolution, Fig. 3).

### Selection of the most robust radiomic features in T1 mapping

We further determined the intersection of features that showed good to excellent repeatability (ICC > 0.75) in all four sequence variations: short-axis and long-axis views, as well as standard and high spatial resolution. This subset of particularly robust features is shown in bold in Fig. 3. In this analysis, we identified 15 features from 6 classes that perform excellent or good in both spatial resolutions and both short-axis and long-axis views.

For these features, we further analyzed for differences in their numerical values between female and male participants as well as younger and older participants, shown in Supplementary Tables 9 and  10 (Supplementary Material). All selected features except sphericity showed significant differences between men and women. For age, no significant differences were seen except for 2 shape features, which may have been confounded by a larger proportion of women in the older age group.

### Repeatability of radiomic features in T2 mapping (standard setting)

T2 mapping acquired in short-axis orientation was considered the clinical standard. For this setting, the repeatability of all radiomic features in each feature class is shown in Supplementary Tables 11–17 (Supplementary Material), sorted in descending order according to their robustness. Substantial differences in the repeatability of radiomic features were seen also for T2 mapping. Across all feature classes, repeatability was excellent for 6 features, good for 25 features, moderate for 23 features, and poor for 46 features. The repeatability of individual features ranged from poor to good or excellent within most feature classes.

### Influence of image orientation on the repeatability of radiomic features in T2 mapping

2D Shape features were the most reproducible, with 77.7% (SAX) and 33.3% (4Ch) of features in this class showing excellent or good reproducibility. All other feature classes showed very limited repeatability, with ≤ 50% of features (SAX) and ≤ 25% of features (4Ch) showing excellent or good repeatability (Fig. 4).

The performances of all features are shown in Fig. 5 for both views. Notably, none of the features in the GLCM feature class showed good or excellent reproducibility for T2 mapping. Figure 6. gives a more detailed representation of those features that showed good to excellent reproducibility in at least one of the views. Overall, the reproducibility of radiomic features was superior in the SAX compared to the 4Ch: 31 features from 6 classes showed good to excellent reproducibility in short-axis T2 mapping, compared to 14 features from 5 classes in the 4Ch (Fig. 6).

### Selection of the most robust radiomic features in T2 mapping

We identified 12 features from 5 classes that showed excellent or good repeatability (ICC > 0.75) in both short-axis and long-axis T2 mapping. This selection of features is shown in bold in Fig. 6.

### Overlap between the most robust radiomic features in T1 and T2 mapping

Seven of the 12 features identified as most reproducible for T2 mapping were also among the 15 most reproducible features for T1 mapping. These include 3 shape features (MinorAxisLength, Maximum2DDiameterSclie, Maximum2DDiameterColumn) and 3 GLRLM features (RunPercentage, RunLengthNonUniformityNormalized, GrayLevelNonUniformity) and 1 GLDM feature (GrayLevelNonUniformity).

## Discussion

Although there is abundant literature on radiomics analysis in cardiac MRI, the majority of these studies have focused on the prognostic and diagnostic value of cardiovascular disease. Radiomics analysis of native T1 and T2 mapping has been applied to myocarditis [5], dilated cardiomyopathy [6, 13, 14], hypertrophic cardiomyopathy [7, 9–11], and myocardial infarction [16], among other pathologies. Only a few of the published papers investigated the reliability of radiomics features in cardiac MRI [19, 30].

In a previous study [30], the repeatability of radiomic features in quantitative T1 and T2 mapping was investigated in 6 healthy participants and 18 patients for the SAX and one resolution. Features with an ICC greater than 0.75 and limits of agreement ± 5% were considered reproducible. Except for 90Percentile, all of the radiomic features found to be most reproducible for T1 mapping in their study also showed good to excellent reproducibility in short-axis standard-resolution T1 mapping in our cohort (Mean, Median, 10Percentile, RootMeanSquared, Imc2, RunLengthNonUniformityNormalized, RunPercentage and ShortRunEmphasis). For short-axis T2 mapping, we can also confirm that the four features identified as reproducible in this previous study (MaximumDiameter, RunLengthNonUniformityNormalized, RunPercentage ShortRunEmphasis), showed good to excellent reproducibility in our cohort in T2 mapping in SAX. In contrast to this previous study, we were able to show that feature robustness varies by resolution (standard resolution or high resolution) and view, resulting in significantly fewer reproducible features for the 4Ch.

Another previous study assessed the repeatability of radiomic features in quantitative T1 and T2 mapping in phantom experiments, 10 healthy participants and 51 patients [31]. In this previous study, features with ICCs greater than or equal to 0.8 were considered reproducible. Consistent with our findings, they also observed that only a minority of radiomic features were reproducible. Several differences between this previous study and ours are noteworthy. The previous analysis [31] focused on the overall performance of feature classes, not individual features, and included an analysis of the influence of image filters. In contrast, our study investigated the repeatability of individual features and the influence of slice orientation (short-axis vs. long-axis view) and spatial resolution on the repeatability of radiomic features. We found that the number of reproducible features was higher for the short-axis than for the long-axis (4Ch) acquisition. This may be related to the orientation of the myocardial muscle fibers relative to the imaging plane or to the geometry of the circular region of interest (ROI) in short-axis mapping compared to the U-shaped ROI in the 4Ch. For spatial resolution, there was no clear trend favoring standard-resolution or higher-resolution maps. We conclude that the standard resolution used in clinical cardiac MR imaging is probably sufficient for radiomic analysis.

While we focused on test–retest repeatability, others have investigated the effect of image processing on radiomic features in T1 and T2 mapping. In patients with hypertrophic cardiomyopathy, Marfisi and colleagues found that image resampling, discretization, and filtering had a significant influence on radiomic features [32]. In their study, radiomic features from T2 maps were less sensitive to changes in image processing than those from T1 maps. A recent study based on a scan-rescan cohort of brain MRI data demonstrated that using a novel physics-informed discretization method substantially improved the number of reproducible radiomic features compared to a conventional fixed bin number discretization [33]. Applying physics-informed discretization methods to image processing in myocardial mapping may thus be a promising avenue to improve the reproducibility of radiomic features.

In our study, we observed that only a limited number of radiomic features demonstrated sufficient reproducibility even though this test–retest study compared two identical MRI examinations performed on the same scanner with identical settings. In clinical practice and cardiovascular imaging studies, cardiac MR examinations are often performed on different scanners with differences in imaging parameters. It is plausible that reproducibility will be even more limited in such a less controlled setting. One previous study found that most radiomic features are indeed sensitive to changes in sequence parameters [22]. For myocardial T1 and T2 mapping, they found that radiomic features were more sensitive to changes in flip angle and slice thickness and less sensitive to changes in in-plane spatial resolution [22]. To address such differences in image acquisition parameters, normalization methods have been developed and shown to improve the robustness of radiomics analysis [34].

In our study, the feature classes 2D shape and GLRLM proved to be more reproducible than other feature classes in our study. Shape features are independent of the gray level intensity distribution in the ROI and are therefore only calculated for the underived image and mask. The segmentations of a randomly chosen subset of participants are shown as an example in Suppl. Fig. 2. Due to the high segmentation agreement by the same radiologist, the agreement between geometric properties of the regions of interest was very high, which is why the 2D shape features predominantly showed good results. The grayscale run length matrix (GLRLM) quantifies the grayscale runs, which are defined as the length in pixels of consecutive pixels with the same grayscale value. The relatively good repeatability in this feature class may be related to the study cohort, which included only healthy subjects with relatively homogeneous T1 and T2 maps. The results may differ in patients with focal or diffuse myocardial disease.

Several of those features that we identified as reproducible have been investigated as diagnostic biomarkers. Among six radiomic features found to discriminate between hypertensive heart disease and hypertrophic cardiomyopathy [11], two GLRLM features (RunLengthNonUniformity and ShortRunEmphasis) were confirmed as highly reproducible in our study. For the diagnosis of acute myocarditis, the combination of two texture features (kurtosis on T2 mapping and GrayLevelNonUniformity on T1 mapping) has been reported as providing the highest diagnostic performance [5]. While GrayLevelNonUniformity on T1 mapping was among the subset of features with high repeatability in our study, we found poor repeatability of kurtosis on T2 mapping. This highlights the need for future radiomic research to focus on features that have been shown to be highly reproducible.

We chose the intraclass correlation coefficient (ICC) as the primary parameter of repeatability in our study. The CCC and Pearson correlation coefficient were used as ancillary parameters to confirm the results. The ICC proposed by Fisher [35], assumes that diagnostic test results follow a one-way ANOVA model with random subject effects and take repeated measurements into account. This model can be extended for replicated measurements and subject-rater interactions, with different versions discussed by Shrout and Fleiss [25]. The CCC proposed by Lin [28] measures agreement without relying on an ANOVA model. The CCC combines consistency (measured by Pearson correlation) and bias, assessing both the closeness of observations to the regression line and the alignment of the regression line to perfect fit [36]. Some authors [29, 36] discuss the limitations of the Pearson correlation coefficient *r* in assessing agreement between test scores, although it can measure the strength of a linear relationship.

In order to check the relationships between these three metrics of reproducibility, they were plotted graphically against each other (Suppl. Fig. 3). The ICC and CCC show a very strong linear relationship. The dependencies of ICC and CCC on the Pearson correlation coefficient *r* also indicate a linear relationship between the metrics with a small degree of scatter.

### Implications for practice

In this work, we have identified 32 radiomics features for short-axis T1 mapping and 15 features for T1 mapping regardless of view and resolution, as well as 12 radiomic features for T2 mapping also independent of view, as particularly robust features with good to excellent repeatability. We recommend these parameters for further research on patients with heart disease. Radiomics capabilities could help distinguish between different heart diseases by revealing subtle differences in tissue texture, morphology, and function that are not visible to the human eye. Another use case could arise for monitoring the success of therapy by using the functions to quantify changes in heart tissue and function during and after treatment. This can help tailor therapy and maximize effectiveness. Nevertheless, further investigation of radiomics data is required to discover new biomarkers and develop new diagnostic and therapeutic approaches. By integrating radiomics capabilities into clinical trials, they can help validate new therapies and improve patient care.

### Study limitations

This prospective study was limited to healthy volunteers. Therefore, we cannot directly infer the reproducibility of radiomic features in patients with diffuse or focal myocardial disease. All maps were calculated using one manufacturer’s 1.5-T MRI scanner Further work is required to confirm the robustness of the recommended radiomic features for T1 and T2 mapping performed with other manufacturers or different field strengths. Likewise, our study was limited to native T1. Without further validation, it is not clear whether the high robustness features also perform equally well for post-contrast T1 maps or extracellular volume (ECV) maps.

## Conclusion

In myocardial T1 and T2 mapping, only a minority of radiomic features have sufficient repeatability to quality as a reliable imaging biomarker. In this study, we have identified a subset of radiomic features that show good to excellent repeatability independent of slice orientation and spatial resolution. We recommend considering these features for further radiomics research in myocardial T1 and T2 mapping.

## Supplementary information

ELECTRONIC SUPPLEMENTARY MATERIAL

## Data Availability

All data are available upon request from the corresponding author.

## Abbreviations

4Ch: Four-chamber view
CCC: Concordance correlation coefficient
GLCM: Gray Level Co-occurrence Matrix features
GLDM: Gray Level Dependence Matrix features
GLRLM: Gray Level Run Length Matrix features
GLSZM: Gray Level Size Zone Matrix features
ICC: Intraclass correlation coefficient
MRI: Magnetic resonance imaging
NGTDM: Neighboring Gray Tone Difference Matrix features
ROI: Region of interest
SAX: Short-axis view

## Code availability

The software source code is available on GitHub (https://github.com/voxelacrobat/Radiomics-on-cardiac-MRI-mapping/).
