# Supplementary material for: Repeatability of radiomic features in myocardial T1 and T2 mapping
Source: Eur Radiol. 2025 Jan 15;35(3):1570–82. doi: 10.1007/s00330-024-11337-8 (PMC11835920; doi:10.1007/s00330-024-11337-8)
Supplement: Supplementary file 1 — ELECTRONIC SUPPLEMENTARY MATERIAL [file 330_2024_11337_MOESM1_ESM.pdf]

Repeatability of radiomic features in myocardial T1 and T2 mapping

ELECTRONIC SUPPLEMENTARY MATERIAL

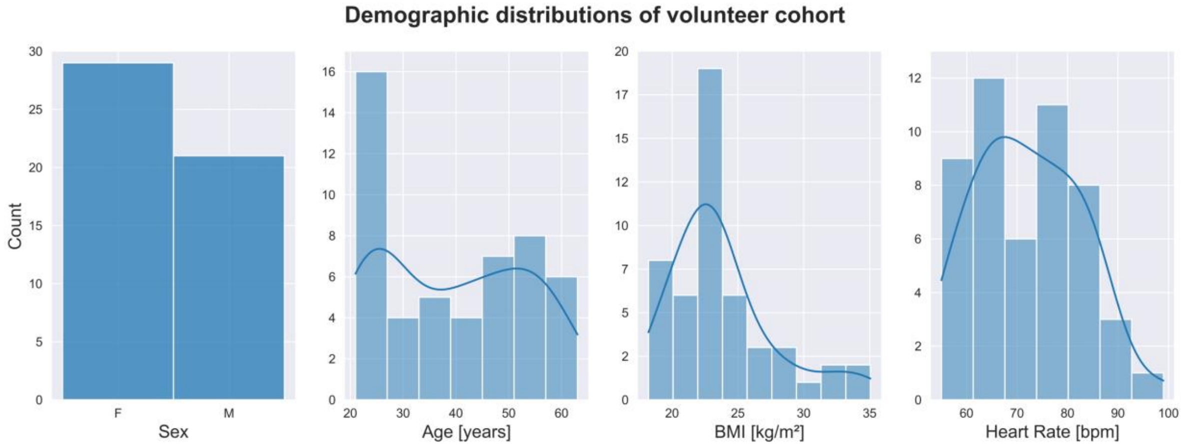

Supplementary Fig. 1: Distributions of volunteer cohort demographic information: *The graphic shows the statistical distributions of age, gender, body mass index (BMI) and heart rate.*

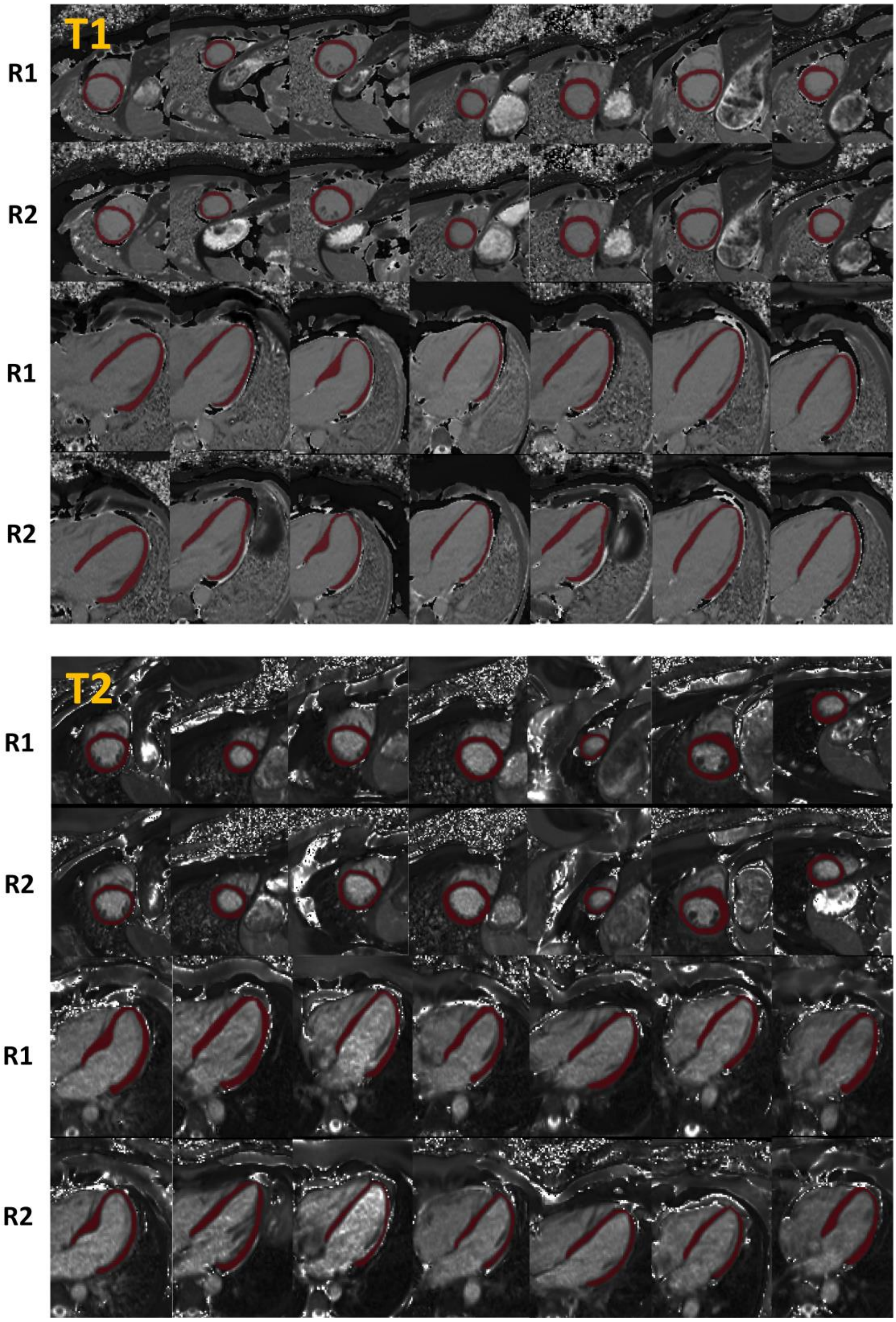

Supplementary Fig. 2 Randomly chosen subset of images with segmentations for T1 and T2 maps: *Random example pairs of images and segmentations for repeat 1 (R1) and repeat 2 (R2), respectively, for short-axis and 4-chamber views, as well as T1 mapping (top) and T2 mapping (bottom).*

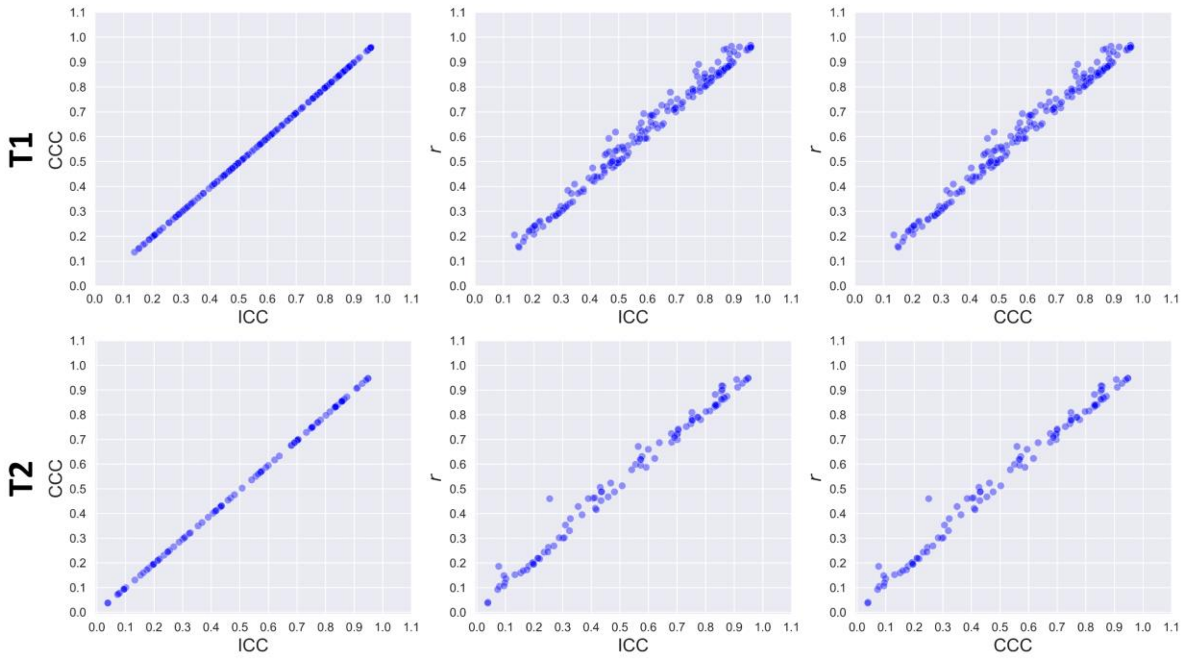

Supplementary Fig. 3: Visual analysis of linear correlation between measures of reproducibility: *The scatter plots show the relationship between the three measures of reproducibility used in our study (ICC, CCC and r) for T1 mapping (top) and T2 mapping (bottom).*

TABLES

Table 1: Complete List of all Radiomic features evaluated.

Index	Feature class	Feature name
1	2D-Shape	original_shape_Elongation
2	2D-Shape	original_shape_Flatness
3	2D-Shape	original_shape_LeastAxisLength
4	2D-Shape	original_shape_MajorAxisLength
5	2D-Shape	original_shape_Maximum2DDiameterColumn
6	2D-Shape	original_shape_Maximum2DDiameterRow
7	2D-Shape	original_shape_Maximum2DDiameterSlice
8	2D-Shape	original_shape_MinorAxisLength
9	2D-Shape	original_shape_Sphericity
10	First_Order	original_firstorder_10Percentile
11	First_Order	original_firstorder_90Percentile
12	First_Order	original_firstorder_Energy
13	First_Order	original_firstorder_Entropy
14	First_Order	original_firstorder_InterquartileRange
15	First_Order	original_firstorder_Kurtosis
16	First_Order	original_firstorder_Maximum
17	First_Order	original_firstorder_MeanAbsoluteDeviation
18	First_Order	original_firstorder_Mean
19	First_Order	original_firstorder_Median
20	First_Order	original_firstorder_Minimum
21	First_Order	original_firstorder_Range
22	First_Order	original_firstorder_RobustMeanAbsoluteDeviation
23	First_Order	original_firstorder_RootMeanSquared
24	First_Order	original_firstorder_Skewness
25	First_Order	original_firstorder_TotalEnergy
26	First_Order	original_firstorder_Uniformity
27	First_Order	original_firstorder_Variance
28	GLCM	original_glcm_Autocorrelation
29	GLCM	original_glcm_JointAverage
30	GLCM	original_glcm_ClusterProminence
31	GLCM	original_glcm_ClusterShade
32	GLCM	original_glcm_ClusterTendency
33	GLCM	original_glcm_Contrast
34	GLCM	original_glcm_Correlation
35	GLCM	original_glcm_DifferenceAverage
36	GLCM	original_glcm_DifferenceEntropy
37	GLCM	original_glcm_DifferenceVariance
38	GLCM	original_glcm_JointEnergy
39	GLCM	original_glcm_JointEntropy
40	GLCM	original_glcm_Imc1
41	GLCM	original_glcm_Imc2
42	GLCM	original_glcm_Idm

43	GLCM	original_glcm_Idmn
44	GLCM	original_glcm_Id
45	GLCM	original_glcm_Idn
46	GLCM	original_glcm_InverseVariance
47	GLCM	original_glcm_MaximumProbability
48	GLCM	original_glcm_SumEntropy
49	GLCM	original_glcm_SumSquares
50	GLRLM	original_glrlm_GrayLevelNonUniformity
51	GLRLM	original_glrlm_GrayLevelNonUniformityNormalized
52	GLRLM	original_glrlm_GrayLevelVariance
53	GLRLM	original_glrlm_HighGrayLevelRunEmphasis
54	GLRLM	original_glrlm_LongRunEmphasis
55	GLRLM	original_glrlm_LongRunHighGrayLevelEmphasis
56	GLRLM	original_glrlm_LongRunLowGrayLevelEmphasis
57	GLRLM	original_glrlm_LowGrayLevelRunEmphasis
58	GLRLM	original_glrlm_RunEntropy
59	GLRLM	original_glrlm_RunLengthNonUniformity
60	GLRLM	original_glrlm_RunLengthNonUniformityNormalized
61	GLRLM	original_glrlm_RunPercentage
62	GLRLM	original_glrlm_RunVariance
63	GLRLM	original_glrlm_ShortRunEmphasis
64	GLRLM	original_glrlm_ShortRunHighGrayLevelEmphasis
65	GLRLM	original_glrlm_ShortRunLowGrayLevelEmphasis
66	GLSZM	original_glszm_GrayLevelNonUniformity
67	GLSZM	original_glszm_GrayLevelNonUniformityNormalized
68	GLSZM	original_glszm_GrayLevelVariance
69	GLSZM	original_glszm_HighGrayLevelZoneEmphasis
70	GLSZM	original_glszm_LargeAreaEmphasis
71	GLSZM	original_glszm_LargeAreaHighGrayLevelEmphasis
72	GLSZM	original_glszm_LargeAreaLowGrayLevelEmphasis
73	GLSZM	original_glszm_LowGrayLevelZoneEmphasis
74	GLSZM	original_glszm_SizeZoneNonUniformity
75	GLSZM	original_glszm_SizeZoneNonUniformityNormalized
76	GLSZM	original_glszm_SmallAreaEmphasis
77	GLSZM	original_glszm_SmallAreaHighGrayLevelEmphasis
78	GLSZM	original_glszm_SmallAreaLowGrayLevelEmphasis
79	GLSZM	original_glszm_ZoneEntropy
80	GLSZM	original_glszm_ZonePercentage
81	GLSZM	original_glszm_ZoneVariance
82	GLDM	original_gldm_DependenceEntropy
83	GLDM	original_gldm_DependenceNonUniformity
84	GLDM	original_gldm_DependenceNonUniformityNormalized
85	GLDM	original_gldm_DependenceVariance
86	GLDM	original_gldm_GrayLevelNonUniformity
87	GLDM	original_gldm_GrayLevelVariance
88	GLDM	original_gldm_HighGrayLevelEmphasis

89	GLDM	original_gldm_LargeDependenceEmphasis
90	GLDM	original_gldm_LargeDependenceHighGrayLevelEmphasis
91	GLDM	original_gldm_LargeDependenceLowGrayLevelEmphasis
92	GLDM	original_gldm_LowGrayLevelEmphasis
93	GLDM	original_gldm_SmallDependenceEmphasis
94	GLDM	original_gldm_SmallDependenceHighGrayLevelEmphasis
95	GLDM	original_gldm_SmallDependenceLowGrayLevelEmphasis
96	NGTDM	original_ngtdm_Busyness
97	NGTDM	original_ngtdm_Coarseness
98	NGTDM	original_ngtdm_Complexity
99	NGTDM	original_ngtdm_Contrast
100	NGTDM	original_ngtdm_Strength

Table 2: Robustness of 2D Shape features in T1 mapping (short-axis view, standard resolution).

**Feature name**	**Robustness**	**ICC (95% CI)**	**CCC**	r
Maximum2DDiameterSlice	excellent	0.960 (0.930, 0.980)	0.959	0.962
MinorAxisLength	excellent	0.944 (0.900, 0.970)	0.943	0.949
Maximum2DDiameterColumn	good	0.868 (0.780, 0.920)	0.866	0.872
MajorAxisLength	good	0.862 (0.770, 0.920)	0.860	0.862
LeastAxisLength	good	0.853 (0.760, 0.910)	0.851	0.852
Sphericity	good	0.799 (0.540, 0.900)	0.796	0.854
Maximum2DDiameterRow	good	0.783 (0.640, 0.870)	0.780	0.796
Flatness	moderate	0.698 (0.520, 0.820)	0.694	0.700
Elongation	poor	0.340 (0.070, 0.560)	0.336	0.339

Table 3: Robustness of First Order features in T1 mapping (short-axis view, standard resolution).

Feature name	Robustness	ICC (95% CI)	CCC	r
TotalEnergy	good	0.900 (0.620, 0.960)	0.898	0.942
10Percentile	good	0.899 (0.830, 0.940)	0.897	0.898
Energy	good	0.886 (0.580, 0.950)	0.884	0.935
Median	good	0.844 (0.730, 0.910)	0.841	0.857
Mean	good	0.822 (0.700, 0.900)	0.819	0.838
RootMeanSquared	good	0.806 (0.680, 0.890)	0.802	0.824
90Percentile	moderate	0.670 (0.470, 0.800)	0.666	0.704
Uniformity	moderate	0.632 (0.350, 0.790)	0.627	0.700
Entropy	moderate	0.612 (0.360, 0.770)	0.607	0.659
InterquartileRange	moderate	0.592 (0.380, 0.750)	0.587	0.619
RobustMeanAbsoluteDeviation	moderate	0.573 (0.350, 0.730)	0.568	0.606
MeanAbsoluteDeviation	moderate	0.563 (0.330, 0.730)	0.558	0.592
Variance	poor	0.476 (0.230, 0.660)	0.471	0.497
Skewness	poor	0.449 (0.200, 0.640)	0.444	0.481
Minimum	poor	0.407 (0.150, 0.610)	0.402	0.424
Maximum	poor	0.201 (-0.050, 0.440)	0.198	0.225
Range	poor	0.174 (-0.080, 0.420)	0.171	0.196
Kurtosis	poor	0.138 (-0.140, 0.400)	0.136	0.205

Table 4: Robustness of Gray Level Co-occurrence Matrix (GLCM) features in T1 mapping (short-axis view, standard resolution).

Feature name	Robustness	ICC (95% CI)	CCC	r
Imc2	good	0.869 (0.780, 0.920)	0.867	0.873
Imc1	good	0.847 (0.750, 0.910)	0.844	0.847
InverseVariance	good	0.758 (0.580, 0.860)	0.754	0.783
Idm	good	0.756 (0.550, 0.870)	0.753	0.792
Id	moderate	0.742 (0.530, 0.860)	0.738	0.778
DifferenceAverage	moderate	0.628 (0.420, 0.770)	0.623	0.651
DifferenceEntropy	moderate	0.601 (0.380, 0.760)	0.597	0.630
Contrast	poor	0.468 (0.230, 0.660)	0.463	0.484
MaximumProbability	poor	0.467 (0.170, 0.670)	0.462	0.540
JointEnergy	poor	0.457 (0.160, 0.670)	0.452	0.532
SumSquares	poor	0.450 (0.200, 0.640)	0.445	0.465
ClusterTendency	poor	0.425 (0.180, 0.620)	0.420	0.439
ClusterShade	poor	0.414 (0.160, 0.620)	0.410	0.420
SumEntropy	poor	0.396 (0.140, 0.600)	0.391	0.434
ClusterProminence	poor	0.368 (0.110, 0.580)	0.363	0.378
DifferenceVariance	poor	0.358 (0.100, 0.570)	0.354	0.372
JointEntropy	poor	0.323 (0.050, 0.550)	0.319	0.385
Correlation	poor	0.305 (0.030, 0.540)	0.300	0.304
JointAverage	poor	0.299 (0.030, 0.530)	0.294	0.320
Idn	poor	0.272 (0.010, 0.500)	0.268	0.282
Autocorrelation	poor	0.223 (-0.040, 0.460)	0.219	0.256
Idmn	poor	0.169 (-0.100, 0.420)	0.166	0.179

Table 5: Robustness of Gray Level Run Length Matrix (GLRLM) features in T1 mapping (short-axis view, standard resolution).

Feature name	Robustness	ICC (95% CI)	CCC	r
GrayLevelNonUniformity	excellent	0.960 (0.930, 0.980)	0,959	0,961
RunLengthNonUniformity	excellent	0.920 (0.600, 0.970)	0,918	0,961
RunPercentage	good	0.884 (0.810, 0.930)	0,882	0,886
ShortRunEmphasis	good	0.882 (0.800, 0.930)	0,880	0,885
RunLengthNonUniformityNormalized	good	0.880 (0.800, 0.930)	0,878	0,882
LongRunEmphasis	good	0.880 (0.800, 0.930)	0,878	0,882
RunVariance	good	0.866 (0.780, 0.920)	0,864	0,867
GrayLevelNonUniformityNormalized	moderate	0.616 (0.330, 0.780)	0,611	0,686
RunEntropy	poor	0.487 (0.220, 0.680)	0,482	0,543
GrayLevelVariance	poor	0.472 (0.230, 0.660)	0,467	0,495
LongRunLowGrayLevelEmphasis	poor	0.294 (0.020, 0.530)	0,290	0,295
LowGrayLevelRunEmphasis	poor	0.284 (0.010, 0.520)	0,280	0,285
ShortRunLowGrayLevelEmphasis	poor	0.281 (0.010, 0.520)	0,277	0,282
LongRunHighGrayLevelEmphasis	poor	0.227 (-0.040, 0.470)	0,223	0,261
HighGrayLevelRunEmphasis	poor	0.208 (-0.060, 0.450)	0,205	0,242
ShortRunHighGrayLevelEmphasis	poor	0.205 (-0.060, 0.450)	0,202	0,239

Table 6: Robustness of Gray Level Dependence Matrix (GLDM) features in T1 mapping (short-axis view, standard resolution).

Feature name	Robustness	ICC (95% CI)	CCC	r
GrayLevelNonUniformity	excellent	0.957 (0.930, 0.980)	0,956	0,958
LargeDependenceEmphasis	good	0.878 (0.800, 0.930)	0,876	0,878
DependenceNonUniformityNormalized	good	0.863 (0.770, 0.920)	0,861	0,867
SmallDependenceEmphasis	good	0.849 (0.750, 0.910)	0,846	0,856
DependenceVariance	good	0.823 (0.710, 0.900)	0,820	0,824
DependenceNonUniformity	good	0.773 (0.390, 0.900)	0,769	0,843
DependenceEntropy	moderate	0.547 (0.270, 0.730)	0,542	0,602
GrayLevelVariance	poor	0.478 (0.240, 0.670)	0,473	0,501
LargeDependenceLowGrayLevelEmphasis	poor	0.450 (0.200, 0.650)	0,445	0,456
LargeDependenceHighGrayLevelEmphasis	poor	0.446 (0.200, 0.640)	0,441	0,479
LowGrayLevelEmphasis	poor	0.290 (0.020, 0.520)	0,286	0,291
SmallDependenceLowGrayLevelEmphasis	poor	0.237 (-0.040, 0.480)	0,234	0,239
HighGrayLevelEmphasis	poor	0.209 (-0.060, 0.450)	0,206	0,243
SmallDependenceHighGrayLevelEmphasis	poor	0.190 (-0.080, 0.440)	0,187	0,222

Table 7: Robustness of Gray Level Size Zone Matrix (GLSZM) features in T1 mapping (short-axis view, standard resolution).

Feature name	Robustness	ICC (95% CI)	CCC	r
GrayLevelNonUniformity	excellent	0.949 (0.890, 0.970)	0,948	0,956
ZonePercentage	good	0.869 (0.780, 0.920)	0,867	0,875
LargeAreaEmphasis	good	0.816 (0.700, 0.890)	0,813	0,818
ZoneVariance	good	0.799 (0.670, 0.880)	0,796	0,803
SmallAreaEmphasis	moderate	0.722 (0.550, 0.830)	0,718	0,739
SizeZoneNonUniformityNormalized	moderate	0.717 (0.550, 0.830)	0,713	0,731
LargeAreaHighGrayLevelEmphasis	moderate	0.635 (0.440, 0.770)	0,630	0,635
SizeZoneNonUniformity	moderate	0.578 (0.240, 0.770)	0,573	0,656
ZoneEntropy	moderate	0.574 (0.360, 0.730)	0,569	0,592
LargeAreaLowGrayLevelEmphasis	moderate	0.517 (0.280, 0.690)	0,512	0,546
GrayLevelVariance	poor	0.416 (0.160, 0.620)	0,411	0,440
GrayLevelNonUniformityNormalized	poor	0.409 (0.140, 0.620)	0,405	0,474
LowGrayLevelZoneEmphasis	poor	0.206 (-0.070, 0.450)	0,202	0,208
HighGrayLevelZoneEmphasis	poor	0.201 (-0.070, 0.440)	0,197	0,233
SmallAreaHighGrayLevelEmphasis	poor	0.188 (-0.080, 0.430)	0,185	0,218
SmallAreaLowGrayLevelEmphasis	poor	0.154 (-0.120, 0.410)	0,152	0,156

Table 8: Robustness of Neighbouring Gray Tone Difference Matrix (NGTDM) features in T1 mapping (short-axis view, standard resolution).

Feature name	Robustness	ICC (95% CI)	CCC	r
Coarseness	good	0.885 (0.730, 0.940)	0,883	0,913
Contrast	moderate	0.691 (0.510, 0.810)	0,687	0,709
Busyness	poor	0.439 (0.180, 0.640)	0,434	0,439
Strength	poor	0.377 (0.120, 0.590)	0,372	0,390
Complexity	poor	0.336 (0.080, 0.550)	0,331	0,372

Table 9: Comparison of best feature ranges between female and male participants in T1 mapping for short axis view and standard resolution

Feature class	Feature name	Female volunteers median (min,max)	Male volunteers median (min, max)	p-value
2D-Shape	MajorAxisLength	77.35 (72.04, 91.24)	86.56 (76.59, 101.11)	<0.005
	Maximum2DDiameterColumn	82.42 (73.63, 89.79)	90.38 (83.08, 97.06)	<0.005
	Maximum2DDiameterSlice	69.49 (60.99, 81.27)	78.56 (69.27, 93.03)	<0.005
	MinorAxisLength	70.57 (65.56, 83.77)	80.37 (72.11, 92.07)	<0.005
	Sphericity	0.28 (0.24, 0.33)	0.32 (0.30, 0.37)	0.4290
GLCM	Imc2	0.85 (0.76, 0.90)	0.72 (0.64, 0.85)	<0.005
GLRLM	ShortRunEmphasis	0.97 (0.96, 0.98)	0.96 (0.94, 0.97)	<0.005
	RunPercentage	0.96 (0.94, 0.97)	0.95 (0.93, 0.96)	<0.005
	RunLengthNonUniformity	727.19 (577.78, 974.41)	1105.45 (835.81, 1497.46)	<0.005
	RunLengthNonUniformity-Normalized	0.93 (0.90, 0.95)	0.90 (0.87, 0.93)	<0.005
	GrayLevelNonUniformity	56.92 (44.88, 91.81)	110.14 (63.43, 140.93)	<0.005
GLSZM	GrayLevelNonUniformity	28.98 (23.92, 40.82)	42.68 (33.24, 56.83)	<0.005
GLDM	GrayLevelNonUniformity	60.78 (46.90, 99.89)	117.85 (67.23, 152.73)	<0.005
First-Order	Energy	9.32E+08 (7.09E+08, 1.23E+09)	1.38E+09 (1.05E+09, 2.01E+09)	<0.005
	TotalEnergy	6.24E+10 (4.36E+10, 8.08E+10)	9.40E+10 (7.13E+10, 1.37E+11)	<0.005

Table 10: Comparison of best feature ranges between younger and older participants in T1 mapping for short axis view and standard resolution

Feature class	Feature name	Younger (≤39 years) Median (min, max)	Older (>39 years) Median (min, max)	p-value
2D-Shape	MajorAxisLength	84.03 (73.61, 101.11)	77.52 (72.04, 94.46)	0.0123
	Maximum2DDiameterColumn	87.84 (74.33, 92.33)	84.67 (73.63, 97.06)	0.2685
	Maximum2DDiameterSlice	75.95 (64.81, 93.03)	69.89 (60.99, 88.96)	0.0198
	MinorAxisLength	79.41 (65.56, 92.07)	73.35 (66.59, 85.65)	0.2859
	Sphericity	0.30 (0.24, 0.37)	0.30 (0.26, 0.37)	0.3319
GLCM	Imc2	0.81 (0.65, 0.90)	0.80 (0.64, 0.89)	0.6554
GLRLM	ShortRunEmphasis	0.97 (0.94, 0.98)	0.97 (0.95, 0.98)	0.7710
	RunPercentage	0.95 (0.93, 0.97)	0.96 (0.93, 0.97)	0.8766
	RunLengthNonUniformity	816.58 (577.78, 1302.58)	835.81 (611.64, 1497.46)	0.6003
	RunLengthNonUniformityNormalized	0.92 (0.87, 0.95)	0.92 (0.88, 0.94)	0.8158
	GrayLevelNonUniformity	79.53 (45.04, 133.09)	72.63 (44.88, 140.93)	0.5605
GLSZM	GrayLevelNonUniformity	37.30 (26.12, 52.48)	33.32 (23.92, 56.83)	0.3516
GLDM	GrayLevelNonUniformity	85.79 (46.90, 147.93)	77.15 (46.96, 152.73)	0.5869
First-Order	Energy	1.07E+09 (7.09E+08, 1.62E+09)	1.08E+09 (7.59E+08, 2.01E+09)	0.5346
	TotalEnergy	7.25E+10 (4.36E+10, 1.10E+11)	7.12E+10 (4.76E+10, 1.37E+11)	0.2859

Table 11: Robustness of 2D Shape Features in T2 mapping (short-axis view).

Feature name	Robustness	ICC (95% CI)	CCC	r
MinorAxisLength	excellent	0.948 (0.910, 0.970)	0.947	0.948
Maximum2DDiameterSlice	excellent	0.928 (0.880, 0.960)	0.926	0.928
Maximum2DDiameterColumn	excellent	0.912 (0.850, 0.950)	0.910	0.912
MajorAxisLength	good	0.874 (0.790, 0.930)	0.872	0.874
LeastAxisLength	good	0.866 (0.780, 0.920)	0.864	0.865
Maximum2DDiameterRow	good	0.859 (0.760, 0.920)	0.856	0.861
Sphericity	good	0.752 (0.470, 0.870)	0.748	0.809
Flatness	moderate	0.622 (0.420, 0.770)	0.617	0.623
Elongation	moderate	0.508 (0.270, 0.690)	0.503	0.513

Table 12: Robustness of First Order features in T2 mapping (short-axis view).

Feature name	Robustness	ICC (95% CI)	CCC	r
TotalEnergy	good	0.855 (0.670, 0.930)	0.852	0.896
Mean	good	0.842 (0.740, 0.910)	0.840	0.841
RootMeanSquared	good	0.835 (0.730, 0.900)	0.832	0.835
Energy	good	0.833 (0.630, 0.920)	0.830	0.882
Median	good	0.831 (0.720, 0.900)	0.828	0.831
Uniformity	good	0.770 (0.620, 0.860)	0.766	0.791
90Percentile	good	0.752 (0.570, 0.860)	0.748	0.785
Entropy	moderate	0.704 (0.500, 0.830)	0.700	0.743
InterquartileRange	moderate	0.702 (0.520, 0.820)	0.698	0.725
10Percentile	moderate	0.681 (0.500, 0.810)	0.676	0.689
RobustMeanAbsoluteDeviation	moderate	0.680 (0.470, 0.810)	0.676	0.724
MeanAbsoluteDeviation	moderate	0.600 (0.350, 0.760)	0.595	0.660
Variance	poor	0.435 (0.180, 0.640)	0.430	0.488
Skewness	poor	0.310 (0.040, 0.540)	0.305	0.353
Minimum	poor	0.133 (-0.150, 0.400)	0.131	0.152
Range	poor	0.102 (-0.180, 0.370)	0.100	0.136
Maximum	poor	0.079 (-0.210, 0.350)	0.077	0.105
Kurtosis	poor	0.076 (-0.200, 0.350)	0.075	0.187

Table 13: Robustness of Gray Level Co-occurrence Matrix features (GLCM) in T2 mapping (short-axis view).

Feature name	Robustness	ICC (95% CI)	CCC	r
JointEnergy	moderate	0.748 (0.600, 0.850)	0.744	0.764
Imc2	moderate	0.701 (0.530, 0.820)	0.696	0.699
Idm	moderate	0.700 (0.520, 0.820)	0.696	0.721
Id	moderate	0.691 (0.510, 0.810)	0.687	0.715
MaximumProbability	moderate	0.691 (0.510, 0.810)	0.686	0.707
JointEntropy	moderate	0.638 (0.410, 0.790)	0.633	0.687
Imc1	moderate	0.592 (0.380, 0.750)	0.587	0.588
SumEntropy	moderate	0.577 (0.330, 0.750)	0.572	0.631
InverseVariance	moderate	0.573 (0.350, 0.730)	0.568	0.595
DifferenceEntropy	moderate	0.571 (0.320, 0.740)	0.566	0.619
DifferenceAverage	moderate	0.555 (0.320, 0.720)	0.550	0.599
Idn	poor	0.434 (0.180, 0.630)	0.429	0.453
Contrast	poor	0.406 (0.150, 0.610)	0.401	0.462
SumSquares	poor	0.390 (0.130, 0.600)	0.385	0.461
ClusterTendency	poor	0.354 (0.100, 0.570)	0.350	0.429
DifferenceVariance	poor	0.327 (0.070, 0.550)	0.322	0.380
Idmn	poor	0.324 (0.060, 0.550)	0.319	0.331
Correlation	poor	0.300 (0.030, 0.530)	0.296	0.300
JointAverage	poor	0.269 (-0.010, 0.510)	0.265	0.269
Autocorrelation	poor	0.181 (-0.110, 0.440)	0.178	0.187
ClusterShade	poor	0.097 (-0.160, 0.350)	0.095	0.120
ClusterProminence	poor	0.039 (-0.230, 0.310)	0.038	0.041

Table 14: Robustness of Gray Level Run Length Matrix (GLRLM) Features in T2 mapping (short-axis view).

Feature name	Robustness	ICC (95% CI)	CCC	r
GrayLevelNonUniformity	excellent	0.948 (0.910, 0.970)	0.947	0.948
RunLengthNonUniformity	excellent	0.908 (0.700, 0.960)	0.907	0.943
LongRunEmphasis	good	0.864 (0.770, 0.920)	0.862	0.870
RunVariance	good	0.855 (0.760, 0.910)	0.852	0.863
RunPercentage	good	0.838 (0.730, 0.900)	0.836	0.840
ShortRunEmphasis	good	0.835 (0.730, 0.900)	0.833	0.836
RunLengthNonUniformityNormalized	good	0.815 (0.690, 0.890)	0.812	0.815
GrayLevelNonUniformityNormalized	good	0.751 (0.580, 0.850)	0.748	0.778
RunEntropy	poor	0.468 (0.190, 0.670)	0.463	0.523
GrayLevelVariance	poor	0.412 (0.150, 0.620)	0.407	0.464
LongRunLowGrayLevelEmphasis	poor	0.305 (0.030, 0.540)	0.301	0.302
ShortRunHighGrayLevelEmphasis	poor	0.215 (-0.070, 0.470)	0.211	0.219
LowGrayLevelRunEmphasis	poor	0.198 (-0.090, 0.450)	0.194	0.195
HighGrayLevelRunEmphasis	poor	0.193 (-0.090, 0.450)	0.190	0.199
ShortRunLowGrayLevelEmphasis	poor	0.175 (-0.110, 0.430)	0.172	0.173
LongRunHighGrayLevelEmphasis	poor	0.095 (-0.190, 0.360)	0.093	0.108

Table 15: Robustness of Gray Level Dependence Matrix (GLDM) Features in T2 mapping (short-axis view).

Feature name	Robustness	ICC (95% CI)	CCC	r
GrayLevelNonUniformity	excellent	0.941 (0.900, 0.970)	0.940	0.942
LargeDependenceEmphasis	good	0.834 (0.720, 0.900)	0.831	0.840
DependenceNonUniformityNormalized	good	0.782 (0.650, 0.870)	0.779	0.780
SmallDependenceEmphasis	good	0.773 (0.630, 0.870)	0.770	0.789
DependenceVariance	good	0.754 (0.600, 0.850)	0.750	0.777
DependenceNonUniformity	moderate	0.704 (0.490, 0.830)	0.700	0.738
LargeDependenceLowGrayLevelEmphasis	poor	0.481 (0.240, 0.670)	0.476	0.488
GrayLevelVariance	poor	0.436 (0.180, 0.640)	0.431	0.489
SmallDependenceHighGrayLevelEmphasis	poor	0.414 (0.160, 0.620)	0.410	0.422
DependenceEntropy	poor	0.368 (0.110, 0.580)	0.363	0.395
LowGrayLevelEmphasis	poor	0.198 (-0.090, 0.450)	0.195	0.195
HighGrayLevelEmphasis	poor	0.196 (-0.090, 0.450)	0.193	0.202
LargeDependenceHighGrayLevelEmphasis	poor	0.095 (-0.190, 0.360)	0.093	0.149
SmallDependenceLowGrayLevelEmphasis	poor	0.038 (-0.250, 0.310)	0.037	0.038

Table 16: Robustness of Gray Level Size Zone Matrix (GLSZM) Features in T2 mapping (short-axis view).

Feature name	Robustness	ICC (95% CI)	CCC	r
LargeAreaEmphasis	good	0.860 (0.770, 0.920)	0.858	0.916
ZoneVariance	good	0.856 (0.760, 0.920)	0.854	0.918
ZonePercentage	good	0.801 (0.670, 0.880)	0.798	0.813
GrayLevelNonUniformity	moderate	0.732 (0.560, 0.840)	0.728	0.752
GrayLevelNonUniformityNormalized	moderate	0.573 (0.320, 0.740)	0.568	0.619
LargeAreaLowGrayLevelEmphasis	moderate	0.564 (0.340, 0.730)	0.559	0.672
ZoneEntropy	poor	0.459 (0.210, 0.650)	0.454	0.468
SizeZoneNonUniformity	poor	0.431 (0.140, 0.640)	0.426	0.506
SmallAreaEmphasis	poor	0.288 (0.020, 0.520)	0.284	0.302
LargeAreaHighGrayLevelEmphasis	poor	0.254 (-0.020, 0.500)	0.250	0.460
SizeZoneNonUniformityNormalized	poor	0.250 (-0.010, 0.490)	0.246	0.263
SmallAreaLowGrayLevelEmphasis	poor	0.248 (-0.030, 0.490)	0.244	0.245
GrayLevelVariance	poor	0.235 (-0.030, 0.470)	0.231	0.244
LowGrayLevelZoneEmphasis	poor	0.220 (-0.060, 0.470)	0.216	0.217
SmallAreaHighGrayLevelEmphasis	poor	0.162 (-0.120, 0.420)	0.160	0.169
HighGrayLevelZoneEmphasis	poor	0.153 (-0.130, 0.410)	0.150	0.160

Table 17: Robustness of Neighbouring Gray Tone Difference Matrix (NGTDM) Features in T2 mapping (short-axis view).

Feature name	Robustness	ICC (95% CI)	CCC	r
Coarseness	good	0.858 (0.680, 0.930)	0.855	0.901
Contrast	moderate	0.541 (0.310, 0.710)	0.536	0.578
Busyness	poor	0.418 (0.160, 0.620)	0.413	0.416
Complexity	poor	0.213 (-0.060, 0.460)	0.210	0.219
Strength	poor	0.073 (-0.210, 0.340)	0.071	0.093
